# Transcriptomic Profiling of Thermotolerant *Sarcomyxa edulis* PQ650759 Reveals the Key Genes and Pathways During Fruiting Body Formation

**DOI:** 10.3390/jof11070484

**Published:** 2025-06-26

**Authors:** Zitong Liu, Minglei Li, Hongyu Ma, Fei Wang, Lei Shi, Jinhe Wang, Chunge Sheng, Peng Zhang, Haiyang Yu, Jing Zhao, Yanfeng Wang

**Affiliations:** 1 Mudanjiang Branch, Heilongjiang Academy of Agricultural Sciences, Mudanjiang 157000, China; lztliuzitong@163.com (Z.L.);; 2 Center of Edible Fungi, Northwest A&F University, Yangling 712100, China

**Keywords:** *Sarcomyxa edulis*, fruiting body development, transcriptome analysis

## Abstract

*Sarcomyxa edulis* is a characteristic low-temperature, edible mushroom in Northeast China. It has a delicious taste and rich nutritional and medicinal value. *S. edulis* can undergo explosive fruiting, neat fruiting, and unified harvesting, making it suitable for factory production. The molecular mechanisms underlying fruiting body development in *S. edulis* remain poorly understood. This study employed transcriptome analysis to compare the post-ripening mycelium (NPM) and primordial fruiting bodies (PRMs) of the thermostable *S. edulis* strain PQ650759, which uniquely forms primordia under constant temperature. A total of 4862 differentially expressed genes (DEGs) (|log2(fold change)| ≥ 1) were identified and found to be predominantly enriched in biological processes such as cell wall organization, DNA replication, and carbohydrate metabolism. KEGG pathway analysis revealed significant enrichment in 20 metabolic pathways, including mismatch repair, yeast cell cycle, and starch/sucrose metabolism. Ten candidate genes (e.g., SKP1, MRE11, GPI) linked to cell cycle regulation, DNA repair, and energy metabolism were randomly selected and prioritized for functional analysis. Quantitative PCR validation confirmed the reliability of transcriptome data, with expression trends consistent across both methods. Our findings provide critical insights into the molecular regulation of fruiting body development in *S. edulis* and establish a foundation for future mechanistic studies and strain optimization in industrial cultivation.

## 1. Introduction

*Sarcomyxa edulis* (Agaricales, Mycenaceae, *Sarcomayxa*) is a well-known low-temperature edible fungus in Northeast China; it is colloquially known as ‘dongmo ‘ or ‘ yuanmo’ [1,2,3]. *S. edulis* is delicate, fragrant, delicious, nutritious, and rich in proteins, polysaccharides, amino acids, and other nutrients [4,5]. It can strengthen muscles and bones, expel wind, and promote blood circulation [6]. Mushroom polysaccharides have anti-tumor, anti-radiation, and immunity-improving effects and high medicinal value [7,8,9]. *S. edulis* is capable of completing its growth and development in a temperature range of 5–20 °C [10,11]. This unique adaptability to low temperatures makes it an important organism in the development of edible fungi resources in the cold regions of the north, but it also limits its cultivation area. As industrial technology has improved, the scale of factory production has also gradually expanded. The short growth cycle and low-temperature characteristics of *S. edulis* make it suitable for factory production. However, at present, the industrial production of *S. edulis* faces multiple technical bottlenecks due to unstable fruiting, strain degradation, and so on. Traditional empirical management relies on phenotypic screening and environmental parameter optimization, but it is difficult to break through the limitations of the genetic potential and metabolic network regulation of the strain. The limitation of traditional empirical management is that farmers rely on experience for management and cannot accurately regulate the cultivation environment, which makes the yield and quality of the fruiting body unstable [2]. Tian et al. [1] sequenced the whole genome of *S. edulis*. Duan et al. [5] conducted transcriptome analysis during the six developmental stages of *S. edulis* and clarified its genetic background. Therefore, it is necessary to understand the growth regulation mechanism of *S. edulis* at the molecular level.

The fruiting body is the most recognizable macrostructure of edible fungi, and it is well known as a source of food and bioactive compounds [12]. In the cultivation of edible fungi, improving the yield and quality of fruiting bodies has always been the most important goal. With the rapid development of the edible fungi industry, the study of fruiting body morphogenesis has become a hot topic [13,14,15]. The process by which basidiomycetes develop into fruiting bodies from mycelia is complex [16]. The life history of basidiomycetes can be divided into two stages: vegetative growth and reproductive growth. As the initial stage of reproductive growth, the primordium stage is an important period affecting the yield and quality of edible fungi [17,18]. In the industrial production of edible fungi, the optimal temperature, humidity, light, and gas conditions for growth can be obtained by artificially regulating environmental parameters, which provides stable and repeatable environmental conditions for edible fungi growth, meaning that production is not limited by region or season [19]. *S. edulis* can undergo explosive fruiting, neat fruiting, and unified harvesting, making it suitable for factory production. However, it is necessary to create a temperature difference of ≥10 °C to form the primordium in the artificially cultivated *S. edulis* strains. During the cultivation process, we found a strain (PQ650759) that can form a primordium under constant temperature conditions. After the optimization of post-ripening and fruiting conditions, the strain can be factory cultivated and year-round production can be realized.

Recent advances in transcriptomic analysis of mushroom-forming fungi have significantly enhanced our understanding of genetic regulation during fruiting body development [20]. Comparative analyses across species such as *Lentinula edodes* has revealed tissue-specific expression patterns, with over 2000 differentially expressed genes (DEGs) identified between dikaryotic mycelia and mature fruiting bodies, highlighting distinct transcriptional reprogramming during morphogenesis [21,22,23,24]. Similarly, stage-specific transcriptional profiling in *Pleurotus tuoliensis* uncovered regulatory genes that were critical for adapting to cultivation parameters [25], while investigations into *Hypsizygus marmoreus* demonstrated size-dependent synergistic interactions among DEGs within niche-specific signaling pathways [26]. The whole genome of *S. edulis* was approximately 35.65 Mb and genome assembly generated 41 contigs with an N50 length of 1,772,559 bp [1]. Building on these findings, the present study focuses on the thermotolerant *S. edulis* strain PQ650759, employing RNA-seq to dissect the transcriptional dynamics between primordial fruiting bodies (PRMs) and vegetative mycelia (NPMs). We deposited the RNA-Seq data in NCBI (PRJNA1274169). Through the integration of DEG screening, KEGG pathway analysis, and qPCR validation, this work identifies conserved and novel genetic drivers—including SKP1, MRE11, and carbohydrate metabolism regulators—that underpin primordium formation under constant-temperature conditions. These insights not only advance our mechanistic understanding of fungal development, but also provide actionable targets for optimizing the industrial-scale cultivation of this economically valuable species.

## 2. Materials and Methods

### 2.1. Mycelial and Fruiting Body Cultures

**Figure 1:**
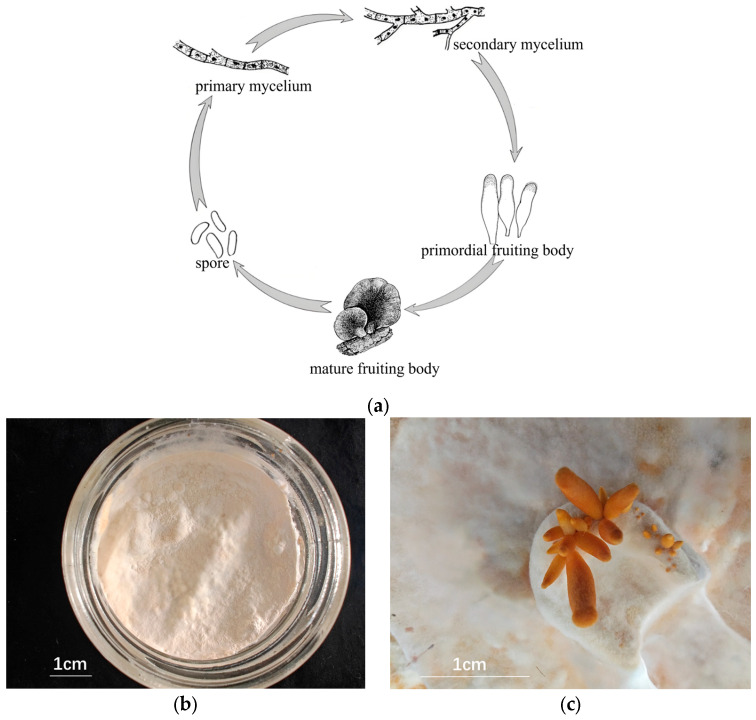
Mycelial and fruiting body cultures of *S. edulis*. (**a**) The life cycle of *S. edulis*; (**b**) non-primordial mycelium (NPM); (**c**) primordial fruiting bodies (PRMs).

The test strain PQ650759 was selected by Mudanjiang Branch, Heilongjiang Academy, and was preserved in the China General Microbiological Collection Center using a sawdust medium formula comprising 86% sawdust, 13% bran, and 1% gypsum. The method for obtaining mycelia and fruiting bodies was as follows: (1) The raw materials were mixed evenly and autoclaved at 121 °C for 40 min. Cellophane was sterilized in advance, and the sterilized cellophane was pasted after the medium was cooled. (2) After inoculation, the culture was maintained at a constant temperature of 25 °C. A total of 60 samples of PQ650759 were cultured for the experiment. (3) When the mycelium was full, it was ripened for 50 days, moved to the mushroom house, and the lid was removed. The temperature of the mushroom house was 16 °C, the humidity was 80%, and the illumination was about 580 Lx. (4) The primordium was partially formed in 15–20 days. Primordial fruiting bodies (PRMs) and non-primordial mycelium (NPM) were obtained, as shown in Figure 1.

### 2.2. Transcriptome Sequencing

#### 2.2.1. Sample RNA Extraction, cDNA Library Construction, and Sequencing

Total RNA was extracted from mycelial and fruiting body tissue using TRIzol^®^ Reagent (Majorbio, Shanghai, China), and the integrity and total amount of the extracted RNA were accurately measured using an Agilent 2100 bioanalyzer (Agilent Technologies, Santa Clara, CA, USA) [27].

The *S. edulis* RNA-seq transcriptome library was prepared following Illumina^®^ Stranded mRNA Prep Ligation (Illumina, San Diego, CA, USA) using 1 μg of total RNA. Briefly, messenger RNA was isolated according to a poly A selection method via oligo(dT) beads and then fragmented using a fragmentation buffer (Illumina, San Diego, CA, USA). Double-stranded cDNA was then synthesized using a Super-Script double-stranded cDNA synthesis kit (Invitrogen, Carlsbad, CA, USA) with random hexamer primers [28]. Then, the synthesized cDNA was subjected to end-repair, phosphorylation, and adapter addition according to the library construction protocol (Illumina, San Diego, CA, USA). Libraries were size-selected for cDNA target fragments of 300 bp on 2% Low Range Ultra Agarose (Invitrogen, Carlsbad, CA, USA) followed by PCR amplification using Phusion DNA polymerase (NEB) (Invitrogen, Carlsbad, CA, USA) for 15 PCR cycles. After quantification using a Qubit 4.0 (Thermofisher, Waltham, MA, USA), the library was sequenced on the Nova-Seq X Plus platform (PE150) using a Nova-Seq Reagent Kit (Illumina, San Diego, CA, USA) [29].

#### 2.2.2. Data Processing and Differential Gene Expression Analysis

The raw paired end reads were trimmed and quality controlled via fastp (ultra-fast all-in-one FASTQ preprocessor) [30], using the default parameters. Then, clean data from the samples were used to perform de novo assembly using Trinity [31]. To increase the assembly quality, all the assembled sequences were filtered using CD-HIT [32] and Trans-Rate [33] and assessed with BUSCO (Benchmarking Universal Single-Copy Orthologs) [34]. The assembled transcripts were searched against the NCBI protein nonredundant (NR), Clusters of Orthologous Groups of proteins (COGs), and Kyoto Encyclopedia of Genes and Genomes (KEGGs) [35] databases using Diamond (http://github.com/bbuchfink/diamond, accessed on 16 June 2025) to identify the proteins that had the highest sequence similarity with the given transcripts to retrieve their functional annotations; a typical E-value cut-off of less than 1.0 × 10^−5^ was set. The BLAST2GO [36] program was used to obtain the GO annotations of unique assembled transcripts for describing biological processes, molecular functions, and cellular components.

To identify DEGs between two different groups, the expression level of each transcript was calculated according to the transcripts per million reads (TPM) method. RSEM [37] was used to quantify gene abundances. Essentially, differential expression analysis was performed using DESeq2 [38]. DEGs with |log2FC| ≧ 1 and FDR < 0.05 (DESeq2) were considered to be significantly differentially expressed genes. In addition, functional enrichment assessments, including GO and KEGG analyses, were performed to identify which DEGs were significantly enriched in GO terms and metabolic pathways at a Bonferroni-corrected *p*-value < 0.05 compared with the whole-transcriptome background. GO functional enrichment and KEGG pathway analysis were carried out using Goatools (https://github.com/tanghaibao/GOatools, accessed on 16 June 2025) and Python scipy software (https://scipy.org/install/, accessed on 16 June 2025), respectively.

#### 2.2.3. Quantitative RT-PCR Validation

The same RNA samples used for the transcriptome analysis were reverse transcribed using HiScript Q RT SuperMix for qPCR (+gDNA wiper Vazyme, Nanjing, China); actin was selected as the internal reference gene. The 20 μL reaction mixture contained 10 μL of 2xTalent qPCR premix (Vazyme Biotech Co., Ltd., Nanjing, China), 0.8 μL of upstream and downstream primers, and 1 μL of cDNA as a template. RNase-free ddH0 was added to the final volume, and qPCR amplification was performed on the ABI stepone real-time PCR system (Applied Biosystems, Fostery, CA, USA). The PCR procedure used in the experiment was 95 °C, 5 min; 95 °C, 30 s; 55 °C, 30 s; 35 cycles; and 72 °C, 1 min.

## 3. Results

### 3.1. Quality Control of Sequencing Data and FPKM

In order to study the mRNA expression changes from mycelium to fruiting body in *S. edulis*, this study used the Illumina sequencing platform to perform transcriptome sequencing on the non-primordial mycelium (NPM) and primordial fruiting body (PRM) of the *S. edulis* strain. The quality of the sequencing data from six samples is summarized in Table 1. The results show that the size of clean reads obtained by filtering the six samples exceeds 6.49 Gb, generating a total of 137 million raw reads. The GC ratio of each treatment was between 51.95% and 52.35%. When evaluating the quality of the original data obtained after removing the low-quality sequences, the percentages of Q20 and Q30 bases in each sample were 98.28 and 94.90% and above, respectively, indicating that the sequencing results were of good quality.

**Figure 2:**
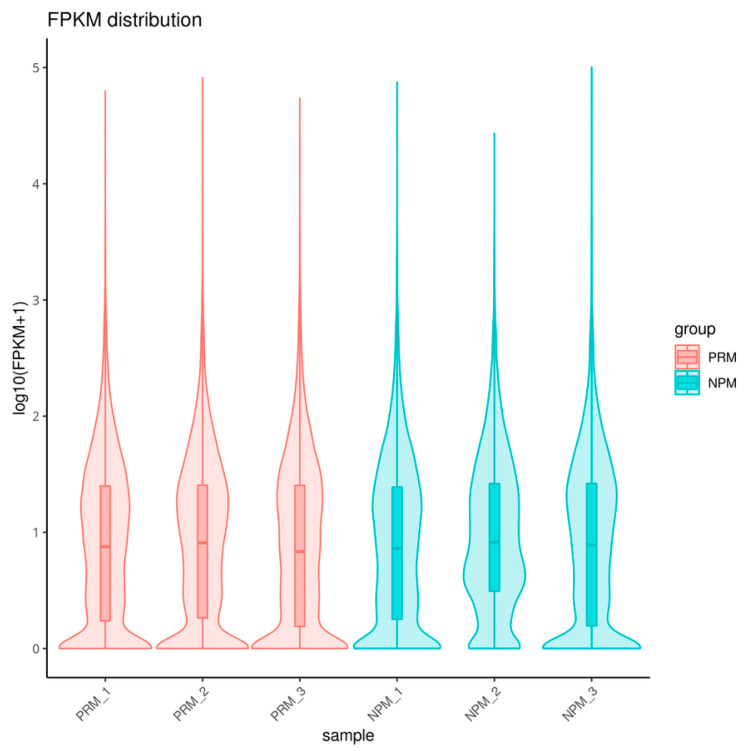
Violin map of the horizontal distribution of gene expression (FPKM) in different PRM and NPM samples. PRM: primordial fruiting body; NPM: non-primordial mycelium. The abscissa is the name of the sample, and the ordinate is log10 (FPKM). The values from top to bottom are the maximum (the topmost position in the longitudinal axis direction), upper quartile (the data value at the 75% position after sorting the data from small to large), median (the vertical axis value corresponding to the position of the middle thick line), lower quartile (the data values at the 25% position after sorting the data from small to large), and minimum (the bottom position in the longitudinal axis direction). The width of each violin represents the number of genes with the same expression.

An FPKM value greater than 1 indicates that the gene is expressed, and higher FPKM values indicate higher expression. The results of gene expression analysis revealed that the median values of the six samples were similar (Figure 2).

### 3.2. Screening of DEGs

**Figure 3:**
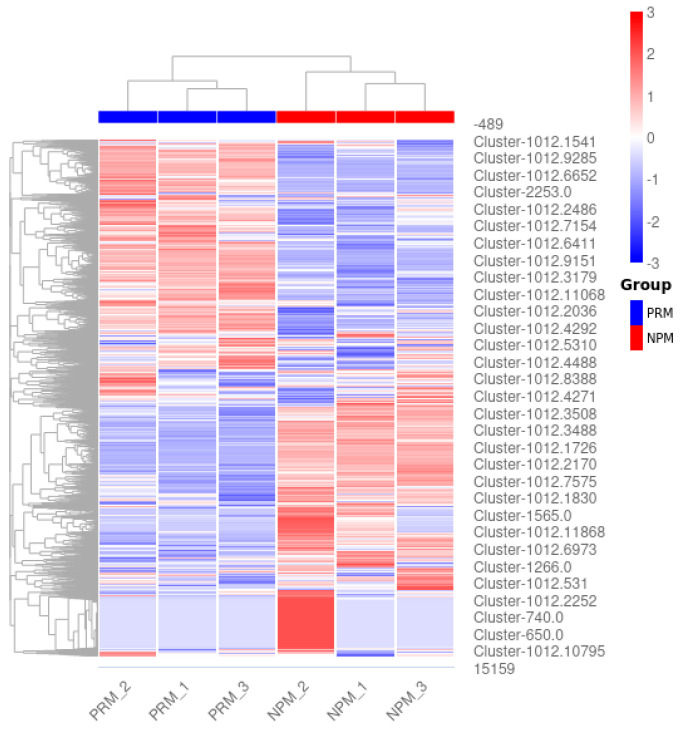
Heat map of different PRM and NPM samples. PRM: primordial fruiting body; NPM: non-primordial mycelium. The abscissa is the sample (PRM and NPM), blue indicates the PRM group, and red indicates the NPM group. The ordinates are different gene clusters and gene numbers. The color on the heat map represents the level of gene expression. The color bar on the right side shows that red indicates high expression, and the larger the value, the redder the color; blue indicates low expression, where the smaller the value, the bluer the color; 0 is the median expression level.

**Figure 4:**
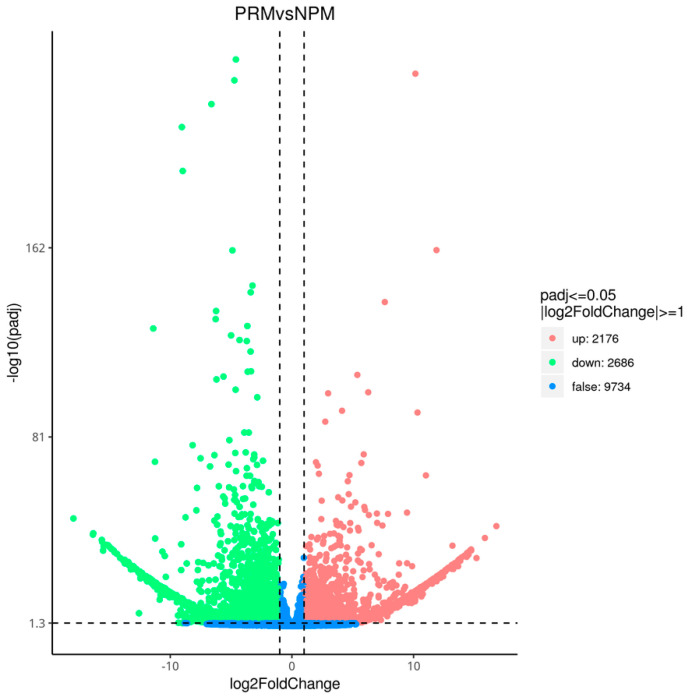
Volcano plots of differentially expressed genes at the PRM and NPM stages. PRM: primordial fruiting body; NPM: non-primordial mycelium. Limits defined by *p*-value ≤ 0.05 and |log2 ratio| ≥ 1. Red points represent significantly upregulated genes; green points represent significantly downregulated genes; the blue area represents insignificant genes.

The transcriptome data from the PRM and NPM were subjected to DEG screening and heat map clustering analysis (Figure 3). The clustering results for the DEGs were significantly different between the two groups; the results for the three replicates in each group were consistent. Volcano plots of the DEGs at the PRM and NPM stages are shown in Figure 4. Red and green represent significantly upregulated and downregulated genes, respectively. The abscissa is the logarithm of the difference in gene expression in the two tissues. The farther away from the origin of the coordinate, the greater the difference. The ordinate is the negative logarithm of the multiple hypothesis test correction for the expression of significantly expressed genes. The larger the ordinate value, the more significant the differential expression of genes and the more reliable the differentially expressed genes screened. There were 4862 genes with a |log2 (fold change)| ≥ 1 between the PRM and NPM, including 2176 upregulated genes and 2666 downregulated genes.

### 3.3. GO Functional Enrichment Analysis of DEGs

**Figure 5:**
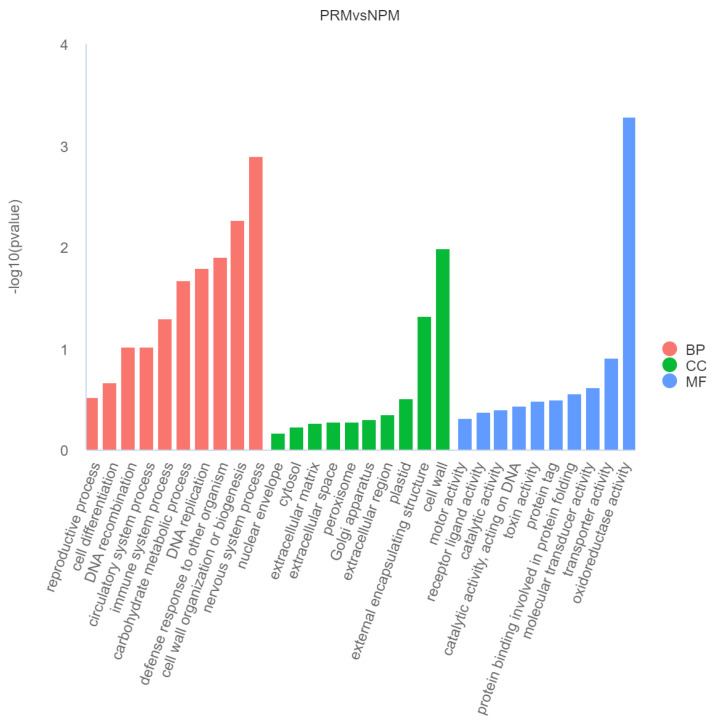
GO enrichment of differentially expressed genes. The red bars represent biological process (BP), the green bars represent cellular component (CC), and the blue bars represent molecular function (MF).

In order to conduct an in-depth analysis of the functions of genes differentially expressed during the fruiting body development of *S. edulis*, GO function enrichment analysis was performed on all differentially expressed genes. The 30 most significant terms from the GO enrichment analysis were plotted on a histogram (Figure 5). The biological functions of the DEGs were described from three aspects: biological process, cellular component, and molecular function. In terms of biological processes, the DEGs were significantly enriched in nervous system process, cell wall organization or biogenesis, defense response to other organisms, DNA replication, carbohydrate metabolic process, and other items. In terms of cellular components, the DEGs were significantly enriched in membrane cell wall, external encapsulating structure, and other items. In terms of molecular functions, the DEGs were significantly enriched in oxidoreductase activity, transporter activity, molecular transducer activity, and other items. The analysis showed that the functions related to carbohydrate metabolic process (GO:0005975), transmembrane transport (GO:0055085), cell wall organization, and biogenesis (GO:0071554) in BP process and oxidoreductase activity (GO:0016491), transporter activity (GO:0005215), and catalytic activity (GO:0003824) in MF process were significantly enriched in downregulated genes during the development from mycelium to fruiting body. The functions related to DNA replication (GO:0006260), telomere organization (GO:0032200), nervous system process (GO:0050877), regulation of transcription (GO:0006355), DNA-templated transcription (GO:0006355), DNA recombination (GO:0006310), DNA repair (GO:0006281), motor activity (GO:0003774), and catalytic activity acting on DNA (GO:0140097) in MF process and the chromosome (GO:0005694) in CC processes were significantly enriched in upregulated genes during the development from mycelia to fruiting body.

### 3.4. KEGG Metabolic Pathway Enrichment Analysis of DEGs

**Figure 6:**
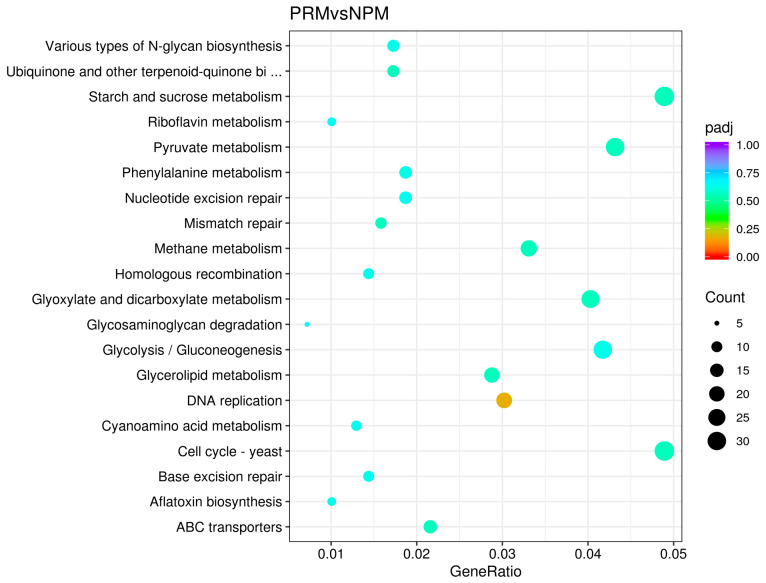
The top 20 enriched KEGG pathways for the DEGs between the PRM and NPM stages. The vertical axis represents the pathway name, and the horizontal axis represents the Gene Ratio corresponding to the pathway. The size of padj (*p*-value adjusted) is represented by the size and color of the bubble. The size of the dot represents the number of differentially expressed genes contained in each pathway.

KEGG is a database that systematically analyzes gene function and connects it to genomic information and functional information. It can effectively reveal the direct relationship between the fruiting body development process and metabolic pathways. All the DEGs in the PRM and NPM samples were annotated in the KEGG database. All the annotated sequences were classified into 20 secondary classification entries, and the enrichment results are shown in Figure 6. The results show that the DEGs were significantly enriched in pathways related to cell proliferation (nucleotide excision repair, mismatch repair, homologous recombination, DNA replication, cell cycle—yeast, base excision repair), energy metabolism (starch and sucrose metabolism, riboflavin metabolism, pyruvate metabolism, methane metabolism, glyoxylate and dicarboxylate metabolism, glycosaminoglycan degradation, glycolysis/gluconeogenesis, ABC transporters), amino acid and pigment synthesis (phenylalanine metabolism, cyanoamino acid metabolism), lipid metabolism (glycerolipid metabolism), and biosynthetic metabolism (various types of N-glycan biosynthesis, ubiquinone and other terpenoid−quinone biosynthesis, aflatoxin biosynthesis).

### 3.5. Analysis of Key Genes for Fruiting Body Occurrence of S. edulis

#### 3.5.1. Genetic Information Processing and Its Key Genes

The development of the fruiting bodies of edible fungi is regulated by a combination of internal regulation and external environmental factors [3,12,39]. Fruiting body formation is affected by multiple factors. The sequential expression of endogenous genes regulates the process of fruiting body formation [40]. In the process of primordium formation from mycelia, the DEGs identified via transcriptome analysis were mainly involved in five types of genetic information processing: cell cycle—yeast, DNA replication, mismatch repair, homologous recombination, and non-homologous end joining. Among them, the S-phase kinase-associated protein 1 (SKP1), DNA replication ATP-dependent helicase/nuclease Dna2, and double-strand break repair protein MRE11 genes were significantly upregulated (Table 1).

#### 3.5.2. Carbohydrate Metabolism and Its Key Genes

Carbohydrate metabolism is one of the most extensive metabolic pathways in plants and includes many biochemical processes. It is the main energy source for mycelial growth and development and has important biological functions. In primordium formation from mycelia, the DEGs identified via transcriptome analysis were mainly involved in four metabolic pathways: glycolysis/gluconeogenesis, starch and sucrose metabolism, the pentose phosphate pathway, and glycosaminoglycan degradation. Among the DEGs, glucose-6-phosphate isomerase (GPI), pyruvate decarboxylase (PDC), 6-phosphogluconate dehydrogenase (PGD), and hexosaminidase were significantly downregulated (Table 1).

#### 3.5.3. Biosynthetic Metabolism and Its Key Genes

When the fruiting bodies of *S. edulis* appear, the DEGs identified from the transcriptome analysis are mainly involved in three types of metabolic pathways: riboflavin metabolism, glyoxylate and dicarboxylic acid metabolism, and the biosynthesis of ubiquinone and other terpenoid−quinone molecules. Among the DEGs, the riboflavin synthase gene was significantly upregulated, while the aconitate hydratase and NAD(P)H dehydrogenase genes were significantly downregulated (Table 2).

### 3.6. qRT-PCR

**Figure 7:**
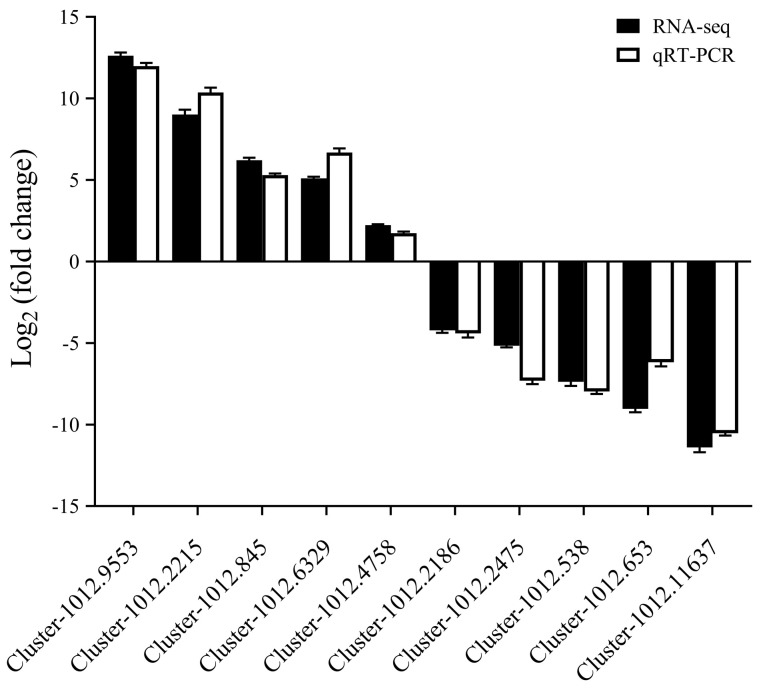
qPCR validation of the differentially expressed genes from Illumina sequencing. Black bars represent the RNA-seq results (log_2_ fold change). White bars represent the qRT-PCR results (2^−ΔΔ*Ct*^).

In order to verify the effectiveness of our RNA-seq data, 10 differentially expressed genes were randomly selected for qRT-PCR analysis. The verification results can be seen in Figure 7. The results from qRT-PCR and RNA-seq data are basically similar and the expression trend is consistent, which fully verifies the reliability of the RNA-seq sequencing results.

## 4. Discussion

The development of edible mushroom fruiting bodies is a complex and rapid process from the formation of the mycelium knot. As the mycelium undergoes structural changes, the primordia of different cell types are formed [16]. In many fungi, primordial development is triggered by various environmental factors, such as nitrogen sources, carbon sources, light, and temperature [41]. At the molecular level, the growth and development of fruiting bodies are accompanied by changes in the expression of related genes, and related biological processes have an important impact on the yield and quality of fruiting bodies [42]. The correlation between gene expression, morphogenesis, and external stimuli is an important factor when predicting gene function [43,44]. During the fruiting body formation in edible fungi, cell proliferation is essential for the differentiation and maturation of fruiting bodies, and the expression of genes will change to meet the needs of growth and development.

As the core component of the SCF complex, SKP1 is involved in the ubiquitination and degradation of substrates such as cyclins [45,46]. During primordium formation, its gene expression was significantly upregulated, providing a material basis for cell cycle progression and the assembly of related protein complexes. Studies have shown that silencing the LeSKP1 gene interferes with the formation of the SCF complex in Lentinula edodes, which mediates the degradation of ARF by AUX/IAA, thereby activating or inhibiting the expression of auxin-responsive genes [47].

The ATP-dependent helicase/nuclease Dna2 uses the energy provided by ATP hydrolysis to unzip double-stranded DNA to form a single-strand template during DNA replication in the fruiting body of S. edulis. This provides a basis for the replication and synthesis of DNA polymerases, ensuring that genetic material can be accurately transferred to progeny cells and ensuring the division and growth of fruiting body cells.

The double-strand break repair protein MRE11 is essential for maintaining genomic stability and normal cell function in organisms [48]. During the transition from vegetative to reproductive growth, genes related to fruiting body formation need to be activated. MRE11 may facilitate the opening of the chromatin region in which these genes are located through chromatin remodeling, which allows for the binding of proteins such as transcription activators and thereby promotes the expression of genes related to fruiting body formation and the development of promoter entities.

SKP1 regulates the ubiquitination and degradation of cyclins through the SCF complex, affects the cell cycle process, and provides a suitable cell cycle environment for CDC20 and MAD2; the regulation of CDC20 and MAD2 in the M phase of the cell cycle will also feed back to the cyclin degradation processes involving SKP1 and jointly maintain the normal cell cycle function [49,50]. Cell proliferation provides a sufficient number of cells and a stable cellular basis for the formation of the fruiting body of *S. edulis*. In eukaryotes, SKP1 interacts with F-box proteins through approximately 40 conserved amino acid residues called F-box sequences [51]. The SKP1 gene plays a key role in the regulation of plant hormone signal transduction. It binds to hormone receptors to form a complex that senses plant hormones, thereby regulating plant growth and development [52,53,54,55,56]. The functions of the SKP1 gene are diverse, and ask1 mutation in Arabidopsis causes male sterility, indicating that the ASK1 gene is essential for the early nuclear recombination of male meiosis [57]. In addition, Nakazawa et al. [58] studied the development regulator gene Cdc5 in *Lentinula edodes* and found that this gene is expressed ubiquitously in the mycelia of immature fruiting bodies; however, in mature fruiting bodies, Cdc5 is specifically expressed in the fruiting layer that forms a large number of basidiospores. Takehito et al. [59] revealed that the transcription of *priB*, *Le.cdc5*, and *ctg1* was activated by low temperatures under appropriate light conditions during the initial stage of fruiting body formation in *Lentinula edodes* FMC2, indicating that temperature-mediated transcriptional regulation may be dependent on the lighting conditions.

In the process of fruiting body formation, Dna2 helicase provides a single-stranded template for the synthesis of the DNA polymerase Pol μ. After the RNA primers are removed by ribonuclease HI, Pol μ can better perform the filling synthesis between Okazaki fragments. MSH2 and MRE11 play roles in mismatch repair and double-strand break repair, respectively. They cooperate with Dna2 and Pol μ to maintain the integrity and accuracy of DNA [60,61,62,63,64,65] and ensure the stable transmission of genetic material from mycelium cells to fruiting bodies.

Glucose-6-phosphate isomerase is one of the key enzymes in glycolysis/gluconeogenesis and plays an important role in cell energy metabolism [66]. When the fruiting body is formed, the metabolic requirements of the cells change and additional substances for the construction of the primordium structure may need to be synthesized. Therefore, the expression and activity of this enzyme are downregulated so that the metabolic flow shifts towards synthetic pathways.

Under aerobic conditions, pyruvate will usually enter mitochondria to participate in the tricarboxylic acid cycle for efficient oxidation and energy supply. However, in the process of primordium formation, cells may need to change their metabolic patterns and reduce energy. In this scenario, pyruvate is used to synthesize other substances, such as amino acids, for cell differentiation and primordium morphogenesis. Therefore, pyruvate decarboxylase is downregulated so pyruvate can be utilized in other metabolic branches.

6-phosphogluconate dehydrogenase is a key enzyme in the pentose phosphate pathway. It catalyzes the dehydrogenation and decarboxylation of glucose-6-phosphate to ribulose-5-phosphate and produces NADPH. NADPH provides an energy supply for cellular anabolism, including the synthesis of fatty and amino acids [67,68]. In the process of fruiting body formation, if the cells focus more on cell division and structure construction, the demand for NADPH for anabolism may be relatively reduced, so the gene is downregulated to adjust the metabolic flux of the pentose phosphate pathway.

Hexosaminidase mainly participates in the degradation of glycosaminoglycans and is capable of hydrolyzing the glycosidic bonds of hexosamine in glycosaminoglycans, gradually degrading glycosaminoglycans into small-molecule substances such as hexosamine [69]. When the fruiting body of *S. edulis* develops from the mycelium, rather than degrading them, the cells may need to use glycosaminoglycans and other substances to construct the extracellular matrix and other structures. Downregulating the hexosaminidase gene can suppress the degradation of glycosaminoglycans, allowing these substances to fulfill their roles in intercellular signal transmission and maintaining the cellular microenvironment, thereby promoting the normal development of the fruiting body.

Lu [70] screened a gene encoded by the carbohydrate-binding module family and three unknown proteins during the development of *Pleurotus ostreatus* fruiting bodies. Wang et al. [71] studied the changes in transcription levels in the mycelia and fruiting bodies of *Agrocybe aegerita*. The results showed that the number of upregulated genes in mycelia and fruiting bodies was 10,131 and 8343, respectively. It is speculated that these genes may be involved in the biosynthesis of sugars and steroids in order to provide sufficient energy for the formation and normal development of fruiting bodies in *Agrocybe aegerita*. In this study, energy-related genes such as glucose-6-phosphate isomerase and glucose-6-phosphate dehydrogenase (PGD) were significantly downregulated. This may be due to modulation of the fungus’s metabolic needs during primordium formation, the shift in cell activities and metabolic pathways to processes related to morphogenesis and tissue differentiation, and the relatively reduced demand for glycolysis and pentose phosphate pathways. Son et al. [72] showed that the pyruvate decarboxylase PDC1 gene is required for lipid accumulation in aerial hyphae, and the deletion of PDC1 leads to a high degree of hyphae wetness.

Riboflavin synthase is mainly responsible for catalyzing the synthesis of riboflavin (VB_2_), and riboflavin plays a very important role in the growth of animals, plants, and microorganisms [73]. During the process of edible fungi developing from mycelium to fruiting bodies, riboflavin synthase ensures an adequate supply of riboflavin within the cells, providing a necessary coenzyme basis for subsequent energy metabolism and redox reactions. In addition, riboflavin upregulation may enhance carotenoid (yellow/orange) or flavin pigment accumulation. This change can be seen from the phenotype of *S. edulis*.

Aconitic acid hydratase is involved in the tricarboxylic acid cycle (TCA cycle), catalyzing the conversion between citric acid and isocitric acid and producing aconitic acid [74,75]. During the development of edible fungi from mycelia to fruiting bodies, aconitic acid hydratase promotes the TCA cycle and provides cells with a large amount of energy (in the form of ATP) and intermediate metabolites.

NAD (P) H dehydrogenase is a key enzyme in the electron transport chain. It catalyzes the transfer of electrons from NAD (P) H to quinones, thereby initiating the transfer of electrons in the respiratory chain, establishing a proton gradient, driving ATP synthesis, and providing energy for cell activity [76].

During fruiting body formation, riboflavin synthase may fine-tune the TCA cycle through a metabolic regulation mechanism, downregulate aconitic acid hydratase, and then catalyze the reaction in the TCA cycle. The NADH produced by the TCA cycle is an important electron donor for the electron transport chain involved in the synthesis/hydrolysis of NAD (P) H dehydrogenase. Changes in all three of these enzymes (riboflavin synthase, aconitic acid hydratase, and NAD (P) H dehydrogenase) together regulate the metabolic and energy flow from the TCA cycle to the electron transport chain in cells, ensuring the balance of various metabolic needs.

The transcriptional verification method is the core technology for analyzing gene expression and function, which is widely used in plant growth and development, stress response, genetic improvement, and other fields [77]. Because rare edible fungi usually have not yet established a stable genetic transformation system, it is impossible to directly verify the function through gene knockout/overexpression. The transcriptional verification method is a key bridge between gene expression data and biological functions. By locating the gene function to construct a regulatory network, it provides a direct target for molecular breeding and cultivation optimization, and further improves the accuracy and systematicness of the research on the development regulation of rare edible fungi.

## 5. Conclusions

The present study focused on the thermotolerant *Sarcomyxa edulis* strain PQ650759, using RNA-seq to dissect the transcriptional dynamics between primordial fruiting bodies (PRMs) and vegetative mycelia (NPMs). By integrating DEG screening, KEGG pathway analysis, and qPCR validation, this work identified conserved and novel genetic drivers—including SKP1, MRE11, and carbohydrate metabolism regulators—that underpin primordium formation under constant-temperature conditions. Functional enrichment analysis revealed pronounced activity in DNA replication, cell cycle regulation, and starch/sucrose metabolism, with the upregulation of SKP1 and MRE11 implicating their roles in genomic stability and cell proliferation. Conversely, the downregulation of glycolytic enzymes (GPI and PDC) suggests a metabolic shift towards structural biosynthesis during fruiting body maturation. These findings not only advance the mechanistic understanding of fungal developmental biology but also provide actionable targets for optimizing the industrial-scale cultivation of this economically valuable species, bridging transcriptomic insights with precision breeding strategies for improved yield and temperature tolerance.

## Figures and Tables

**Table 1:** Sequencing data quality summary for PRM and NPM.

Sample	Raw Reads	Clean Reads	Error Rate	Q20	Q30	GC pct
PRM1	22749365	208998714	0.01	98.31	94.99	52.10
PRM2	24370641	22972599	0.01	98.34	95.12	51.97
PRM3	21640785	20346185	0.01	98.28	94.90	51.99
NPM1	22260169	21646551	0.01	98.36	95.20	52.35
NPM2	23581987	21386247	0.01	98.46	95.41	52.15
NPM3	22721561	21143993	0.01	98.36	95.18	51.95

PRM: primordial fruiting body; NPM: non-primordial mycelium. Q20: the percentage of bases with Phred values greater than 20 in the total bases; Q30: the percentage of bases with Phred values greater than 30 in the total bases; GC: the ratio of the number of G and C bases to the total number of bases in clean reads after filtering.

**Table 2:** RNA-seq data of selected 10 key genes.

Gene Name	Gene ID	log2FoldChange	*p* Value	padj
S-phase kinase-associated protein 1	Cluster-1012.804	5.6367	2.54 × 10^−3^	6.89 × 10^−3^
DNA replication ATP-dependent helicase/nuclease Dna2	Cluster-1012.804	3.6928	3.19 × 10^−7^	1.69 × 10^−6^
Double-strand break Repair protein MRE11	Cluster-1012.4758	2.2675	1.07 × 10^−16^	1.65 × 10^−15^
Glucose-6-phosphate isomerase	Cluster-1222.0	−7.3875	5.33 × 10^−3^	1.34 × 10^−2^
Pyruvate decarboxylase	Cluster-1012.7332	−2.0115	1.75 × 10^−25^	5.74 × 10^−24^
6-Phosphogluconate dehydrogenase	Cluster-1012.2679	−6.0057	5.99 × 10^−63^	1.35 × 10^−60^
Hexosaminidase	Cluster-1012.10120	−3.1094	1.59 × 10^−15^	2.19 × 10^−14^
Riboflavin metabolism	Cluster-1012.6329	5.1008	1.40 × 10^−17^	2.37 × 10^−16^
Aconitate hydratase	Cluster-1012.319	−7.9724	1.84 × 10^−3^	5.17 × 10^−3^
NAD(P)H dehydrogenase	Cluster-1012.8236	−2.4582	8.41 × 10^−8^	4.83 × 10^−7^

## Data Availability

The data presented in this study are openly available at NCBI at http://www.ncbi.nlm.nih.gov/bioproject/1274169, accessed on 10 June 2025, reference number PRJNA1274169.
